# Childhood vaccination practices and associated factors among mothers/caregivers in Debre Tabor town, Northwest Ethiopia: A cross-sectional study

**DOI:** 10.3389/fped.2023.1070722

**Published:** 2023-01-30

**Authors:** Wudneh Simegn, Mengistie Diress, Yibeltal Yismaw Gela, Daniel Gashaneh Belay, Anteneh Ayelign Kibret, Dagmawi Chilot, Deresse Sinamaw, Mohammed Abdu Seid, Amare Agmas Andualem, Desalegn Anmut Bitew, Habitu Birhan Eshetu, Abdulwase Mohammed Seid

**Affiliations:** ^1^ Department of Social and Administrative Pharmacy, School of Pharmacy, College of Medicine and Health Sciences, University of Gondar, Gondar, Ethiopia; ^2^ Department of Human Physiology, School of Medicine, College of Medicine and Health Sciences, University of Gondar, Gondar, Ethiopia; ^3^ Department of Human Anatomy, School of Medicine, College of Medicine and Health Sciences, University of Gondar, Gondar, Ethiopia; ^4^ Department of Epidemiology and Biostatistics, Institute of Public Health, College of Medicine and Health Sciences, University of Gondar, Gondar, Ethiopia; ^5^ Center for Innovative Drug Development and Therapeutic Trials for Africa (CDT-Africa), Addis Ababa University, College of Health Sciences, Addis Ababa, Ethiopia; ^6^ Department of Biomedical Science, Debre Markos University, Debre Markos, Ethiopia; ^7^ Unit of Human Physiology, Department of Biomedical Science, College of Health Sciences, Debre Tabor University, Debre tabor, Ethiopia; ^8^ Department of Anesthesia, Wollo University, Dessie, Ethiopia; ^9^ Department of Reproductive Health, Institute of Public Health, College of Medicine and Health Sciences, University of Gondar, Gondar, Ethiopia; ^10^ Department of Health Promotion and Health Behavior, Institute of Public Health, College of Medicine and Health Sciences, University of Gondar, Gondar, Ethiopia; ^11^ Department of Clinical Pharmacy, School of Pharmacy, College of Medicine and Health Sciences, University of Gondar, Gondar, Ethiopia

**Keywords:** childhood vaccination, practices, associated factors, mothers/caregivers, Ethiopia

## Abstract

**Background:**

Incomplete immunization and non-immunization increase the risk of disease and death among children. This study aims to assess childhood vaccination practices and associated factors among mothers and caregivers in Debre Tabor town, Amhara region, Ethiopia.

**Methods:**

A community-based cross-sectional study design was conducted between February 30 and April 30, 2022. The study participants were proportionally allocated to all six kebeles found in the town. A systematic random sampling technique was used to select the study participants. The collected data were checked and coded and then entered into EpiData Version 3.1 and exported into SPSS Version 26. The results were organized using frequency tables, graphs, and charts, and bivariate and multivariable logistic regression were used to test the association of covariates with childhood vaccination practices.

**Result:**

Approximately 422 study mothers and caregivers participated in the study, with a response rate of 100%. The mean age was 30.63 years (11.74), which ranged from 18 to 58 years. More than half of the study participants (56.4%) expressed fears about the side effects of vaccination. A majority (78.4%) of the study participants availed of counseling services about vaccination, and 71.1% of them received regular antenatal care. This study found that approximately 280 [66.4%, 95% confidence interval (CI): 61.8–70.6] mothers/caregivers had a history of good childhood vaccination practices. The factors of the fear of side effects [adjusted odds ratio (AOR) = 3.34; 95% CI: 1.72–6.49], no workload (AOR = 6.08; 95% CI: 1.74–21.22), medium workload (AOR = 4.80; 95% CI: 1.57–14.71), being a mother of child/children (AOR = 2.55; 95% CI: 1.27–5.13), positive attitude (AOR = 2.25; 95% CI: 1.32–3.82), and sound knowledge (AOR = 3.88; 95% CI: 2.26–6.68) were significantly associated with childhood vaccination practices.

**Conclusion:**

More than half of the study participants had a history of good childhood vaccination practices. However, the rate of such practices was low among mothers and caregivers. The fear of side effects, workload, motherhood, attitude, and knowledge were all factors associated with childhood vaccination practices. Awareness creation and a consideration of the workload of mothers would be helpful in dispelling fears and increasing the rate of good practices among mothers and caregivers.

## Introduction

Vaccination is considered an important community health intervention that helps prevent vaccine-related diseases, in turn, preventing childhood morbidity and mortality (1–4). Vaccination is the most cost-effective public health intervention and disease prevention method (5). Therefore, immunization efforts have been strengthened worldwide (6, 7). Currently, the Expanded Program on Immunization (EPI) program provides 11 antigens aimed at preventing major childhood killer diseases occurring during the child's first year of life (4). The EPI in Ethiopia, which was started in the year 1980 of the last century, is one of the main components of the current Health Sector Plan (HSP) of the country (4). This health service has been significantly improved to achieve full vaccination coverage between the years 2000 and 2016 (8) of the present century.

More than 35% of World Health Organization (WHO) member nations, including Ethiopia, are devising plans to reach the 90% coverage mark for the third dose of diphtheria, tetanus, and pertussis-containing vaccine (9). Even though there have been incredible efforts to provide full vaccination coverage, the rates are very low in east Africa (10). Such low rates point to the need for taking effective steps to engage caregivers in immunization programs in a better way, while also addressing the yawning gaps in the health system (11, 12).

The WHO had received reports of over 4,000 suspected cases of diphtheria and 30 diphtheria-related deaths by January 2018 (13). In many countries, including Ethiopia, full immunization coverage was not being achieved and immunization staff often tried to assess changes over time (14). A systematic review and meta-analysis showed that the pooled full immunization coverage using the random-effect model was 58.92% in Ethiopia (15).

Because of barriers in immunization service utilization, a large proportion of children in many countries fail to benefit from the positive effects of all basic vaccines and vaccine-preventable diseases, with the highest rates of child mortality reported in sub-Saharan Africa, including Ethiopia (4, 16). Previous studies in Ethiopia indicate that mothers completing the recommended antenatal care (ANC) appointments were eligible to receive at least one dose of vaccination, and maternal education was associated with complete vaccination services (17). According to a qualitative study conducted in Hadiya zone, Ethiopia, the main reason for immunization failure was a lack of counseling of mothers, which resulted in a lack of information about vaccination plans and service schedules, leading to missed appointments and loss of vaccination cards when health workers failed to make home visits (18). Another study showed that children from poor households and the absence of maternal education in such households were responsible for low vaccination coverage (19). In Dessie town, Ethiopia, researchers discovered that associations between ANC follow-up, parental vaccine knowledge, the mother’s education level, and family size served as the main factors behind poor vaccination practices (20). A cross-sectional study in Northwest Ethiopia also showed that regular antenatal care and delivery provided in a facility was associated with good child vaccination practices (21). A cross-sectional study in Debre Markos discovered that children’s gender, regular ANC follow-up, and distance from the health facility were factors associated with good childhood vaccination practices (22). According to a cross-sectional study conducted in Worabe, Ethiopia, the most common reasons for parents not vaccinating a child are the fear of side effects (36%), being too busy at work (31%), and a lack of time (13%) (13). Another cross-sectional study, this time in India, showed that being busy, poor knowledge, fear of side effects, poor daily income, and migrant population were the major reasons for not availing immunized services (23). A review paper revealed that hindrances to vaccination were concerns about vaccine side effects, a lack of trust, social norms, and a lack of facility access. Sociodemographic factors such as sex of the child, birth order, mothers’ educational status, monthly income of parents, and religion were reported to be associated factors of good childhood vaccination practices (24).

To successfully control vaccine-preventable diseases, adequate vaccination coverage should be provided to meet the WHO target of 90% coverage across the world (4). To achieve this target, updated research output about childhood vaccination practices and the associated factors is important to design appropriate interventions by the relevant stakeholders. Therefore, this study aims to assess childhood vaccination practices and their associated factors among mothers and caregivers in Debre Tabor town, Northwest Ethiopia.

## Methods

### Study design, setting, and period

A community-based cross-sectional study was conducted in Debre Tabor town, Amhara Region, Ethiopia. It is located in the South Gondar zone, Northwest Ethiopia, 99 km away from Bahir Dar City and 667 km away from Addis Ababa, the capital of Ethiopia. There are six kebeles in the town with a total population of 85,727, of which 49.6% (42,521) were men and 50.4% (43,206) were women. There were 19,936 households based on information obtained from the town health office for the year 2020 (25). The study period was from February 30 to April 30, 2022.

### Population

The source of the population was all mothers and caregivers in Debre Tabor town with children under the age of 5. Randomly selected mothers or caregivers living in the town who fulfilled the eligibility criteria formed the study population.

### Inclusion and exclusion criteria

All mothers or caregivers having children between the age of 2 months and 5 years, greater than 18 years old, having the required consent to participate, and available at their homes during the study period were included in the study. Those who had psychiatric illness during the study period were excluded.

### Study variables

The dependent variable in this study is childhood immunization practice and the independent variables are as follows: sociodemographic characteristics of mothers or caregivers such as age, religion, educational status, marital status, occupation, average monthly income, number of children under 5 years, number of families living together, sex of the smallest child in the family, age of the smallest child in months, relationship with the child (mother or caregiver), whether availing the benefits of home visits by health professionals, whether receiving counseling about childhood vaccination, whether taking regular antenatal care during pregnancy, the distance of the home from the health facility, the source of information about childhood vaccination, workload, fear of side effects of vaccination, if any, and reasons for missing childhood vaccination.

### Sample size determination and sampling technique

The sample size was determined using a single population proportion formula and the prevalence of the previous study (55.3%) (26) with the assumption of a 5% margin of error and a 95% confidence level. By adding a 15% non-response rate, the final sample size was found to be 435 mothers or caregivers with their children.


$$n=\frac{{\left(\;z_{\frac{a}{2}}\;\right)}^{2}\times p\times \left(1-p\right)}{d^{2}}=\frac{{\left(1.96\right)}^{2}\times 0.553\times \left(1-0.553\right)}{{\left(0.05\right)}^{2}}=379$$

Considering a 15% non-response rate, the final sample totaled 435.

**Figure 1:**
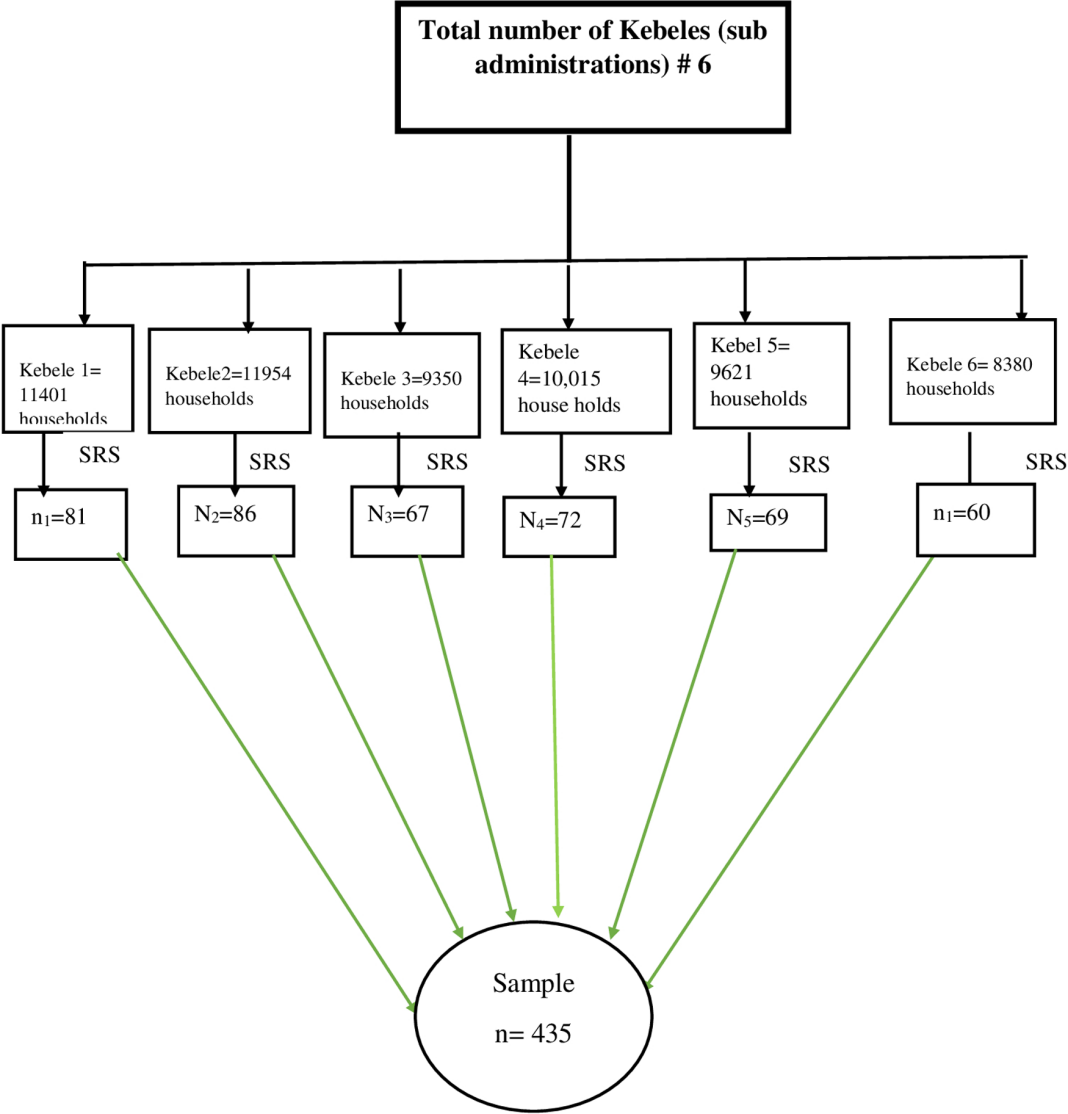
Schematic presentation of the sampling procedure for the assessment childhood vaccination practices among mothers/care givers, 2022 (*n* = 422).

The number of households having children was calculated by considering each kebele administrative office. The sample size was allocated proportionally to each of the six kebeles. The samples of the households in each of the kebeles were determined by dividing the total number of households having children by the allocated sample size (Figure 1).

### Data collection instrument

The data collection instrument used for this study resembled that of previous similar studies (16, 24, 26–30). The first part of the study included sociodemographic and related questions consisting of age, religion, marital status, educational status, occupation, average monthly income, number of children under 5 years, number of families living together, sex of the smallest child in the house, age of the smallest child in months, relationship with the child (mother or caregiver), whether availing the benefits of home visits by health professionals, whether receiving counseling about vaccination, whether taking regular antenatal care, home distance from a health facility, whether knowing the source of information about vaccination, workload, and the fear of the side effects of vaccination.

The second part of the study included the 12 knowledge items: vaccination is important for children from the first day of birth; vaccination prevents infectious diseases; vaccination reduces death and disability; vaccination keeps children healthy; diphtheria, tetanus, and pertussis are controlled through vaccinations; hepatitis B virus can be prevented by vaccination; childhood vaccinations control measles; malnutrition, low fever, and diarrhea are not contraindications to vaccination; some vaccinations are related to fever and pain; vaccinations may cause cramps and rashes; even a healthy child needs vaccinations; and it is necessary to vaccinate a breastfeeding infant.

The third part included eight items of attitude: Do you think vaccinations are beneficial; do you feel that it is safe to have your child vaccinated; do you think compliance with the immunization schedule is important; do you think vaccination side effects are dangerous; do you think vaccination is important only for non-serious diseases; do you think vaccination makes infants vulnerable to disease and death; do you think all children should be vaccinated; and do you think vaccination makes infants sick?

The fourth part included eight items to measure the practice: Has your child received the recommended childhood vaccines until the current age; do you follow the compulsory vaccination programs listed in the vaccination schedule; do you use pain relievers to relieve swelling and pain after having your child vaccinated; are you given information about the current vaccine; did the healthcare worker tell you the type of vaccine your infant has taken; did the healthcare worker tell you the dose of the vaccine your infant has taken; do you know when the next vaccination date is for your infant; and do you know about the side effects of EPI vaccines.

Forward and back translation procedures were performed. The principal investigator prepared all items that measured the knowledge, attitudes, and practices related to childhood vaccination. One expert carried out the translation from English to Amharic. This forward translation was then translated by a second expert back to Amharic. There was no difference between the two English versions.

### Data collection procedure

Interview-based structured questionnaires were administered to collect information from mothers or caregivers by well-trained interviewers. The initial interviewed mothers/caregivers were selected by the lottery method from the sampling interval using a number between 1 and the sampling interval. After selecting the first mother or caregiver, the subsequent mothers or caregivers were selected using the systematic random sampling technique. If no respondents were found in the planned mother or caregiver group, a nearby mother or caregiver was interviewed until the required sample size was reached. The data collectors contacted the mothers/caregivers for interview. If the mothers/caregivers had two and more than two children between 2 months and 5 years, the data collector focused on the smallest child to fill the questionnaire. Four data collectors (BSc nurses) and two supervisors were recruited based on a set of criteria such as knowledge of the Amharic language and previous experience in data collection. The principal investigator was imparted a 1-day training on the purpose of the study, data collection tools or instruments, how to conduct the interview and how to obtain information, and the overall data collection procedures. In order to reduce recall bias, different recalling techniques such as routes of administration (checking injection sites and the presence of a scar on the arm) were used.

### Statistical analysis

Data collected from the survey was entered using EpiData and exported to statistical data analysis software (Statistical Package for Social Science, SPSS Version 26). Descriptive statistics were computed for mean, frequency, and percentage. The multiple logistic regression model was used to identify the associated factors. Odds ratios (ORs) and their 95% confidence intervals (CIs) were calculated. Then, all variables that had a *P*-value of less than 0.2 in the bivariate analysis were included in the multiple logistic regression analysis models to determine the factors associated with knowledge, attitude, and practice. A variable with a *P*-value of less than 0.05 was declared to be associated with the outcome variable. The scales’ reliability was assessed using Cronbach's alpha in the pilot study. A Cronbach's alpha coefficient greater than 0.70 was considered acceptable (28).

### Pilot study

The authors conducted a pilot test on 30 study participants outside of the study area (Gondar Town) to evaluate the clarity and reliability of the tool. Based on the pilot test, the authors amended a few questions and omitted one item from the attitude section and two items from the practice section.

### Quality control

The Cronbach’s alpha coefficient in the pilot study was *α* = 0.77, *α* = 0.81, and *α* = 0.79 for knowledge, attitude, and practice, respectively. This indicated that the tools for measuring knowledge, attitude, and practice were highly reliable. With regard to validity, the instrument used was the same one used in the previous local study. The principal investigator provided training to the supervisors and data collectors about the objectives, methods, and instrument of the study, the ethical principles of the study, and data collection procedures. The data collectors submitted the collected data every day to the supervisors to check the completeness and consistency of the data.

### Operational definition

**Knowledge:** To measure the knowledge of the participants, 12 items with true/false answers were used. One point was given for the correct answer, and zero was given for the incorrect answer. Three items (items 202, 205, and 211) were reverse-scored; that is, the participants would have 1 point if their answer was false. The sum was dichotomized as good knowledge and poor knowledge using the mean score of the data (6.0). Study participants who scored the mean and above the mean score (the data were normally distributed) were considered to have good knowledge of childhood vaccination practices, while those who scored below the mean were considered to have poor knowledge of such practices (26).

**Attitude:** Eight Likert scale questions with three point scales (agree, neutral, and disagree) were used to measure the attitude of the study participants toward childhood vaccination. The scoring system was as follows: agree = 2, neutral = 1, and disagree = 0, and the score was reversed for negative end questions (four questions, 4th, 5th, 6th, and 8th). The total score of attitude ranged from 0 to 16 points, and the mean score was 8.0. Study participants who scored the mean or more than the mean score were considered to have a positive attitude toward childhood vaccination practices, while those scoring less than the mean were considered to have a negative attitude toward childhood vaccination practices as in the previous study (26).

**Practice:** Eight items with “yes, no, and I don’t know” answers were used to measure the practices of the study participants. The point for the correct answer was 1; for the incorrect answer and for those who responded with “I don’t know,” 0 points were given. The mean score was 4.0, and those study participants who scored the mean and more than the mean (the data were normally distributed) were considered to have good childhood vaccination practices, while those who scored less than the mean were considered to have poor practices (26).

### Ethical clearance

Ethical clearance was obtained from the School of Pharmacy, University of Gondar. The participation of the respondents was voluntary with verbal informed consent after a detailed explanation of the purpose of the study. Participants’ responses were anonymous, and data collectors informed them that they had the complete right to discontinue or refuse to participate in the study. The participants were informed that the collected data were used for research purposes only.

## Result

### Sociodemographic characteristics

In this study, 422 mothers and caregivers participated, with a response rate of 97%, and even all caregivers were women. The mean age was 30.63 ± 11.74 years, which ranged from 18 to 58 years. The majority (61.8%) of them were followers of the Orthodox Church. Approximately 107 (25.4%) participants had a college or above level of education, and the majority (57.6%) were married. Approximately 211 (50.0%) were housewives, followed by government employees (20.4%) (Table 1).

**Table 1:** Sociodemographic characteristics of study participants among mothers/caregivers in Debre Tabor, Northwest Ethiopia, 2022 (*n* = 422).

Variable	Categories	Frequency	Percent
Age in years	18–27	106	25.1
	28–33	109	25.8
	34–42	107	25.4
	43–58	100	23.7
Religion	Orthodox	261	61.8
	Protestant	48	11.4
	Catholic	16	3.8
	Muslim	97	23.0
Educational status	Illiterate	96	22.7
	1–8	108	25.6
	9–12	104	24.6
	College and above	107	25.4
Marital status	Single	179	42.4
	Married	243	57.6
Occupation	No work	70	16.6
	Governmental	86	20.4
	Housewife	211	50.0
	Private	55	13.0
Average monthly income in Ethiopian Birr (ETB)	<2000	120	28.4
	2001–4000	92	21.8
	4001–6000	105	24.9
	6001+	105	24.9

### Vaccination-related information of study participants

Most of the study participants (76.1%) had only one child. Approximately 82.2% of them were mothers and 17.8% of them were caregivers (not mothers for the child). The majority of the study participants (56.4%) expressed fears about the side effects of vaccination. Three hundred and twenty-one (76.1%) participants had only one child under five, and a few participants (5.9%) were living only with their one child. In this study, 78.4% of study participants had received counseling services about vaccination, and 71.1% took regular antenatal care. Approximately 56.4% of mothers and caregivers reported about the fears of the side effects of childhood immunization. In this study, approximately two-thirds (62.1%) had good knowledge of childhood vaccination practices and more than half (54.7%) had a positive attitude toward such practices (Table 2).

**Table 2:** Vaccination-related information of study participants among mothers/caregivers in Debre Tabor, Northwest Ethiopia, 2022 (*n* = 422).

Variable	Categories	Frequency	Percent
Number of children under 5 years	Only one child	321	76.1
	Two children	101	23.9
Number of families living together	Only two with child/children	25	5.9
	Three with child/children	102	24.2
	Four with child/children	141	33.4
	Five and above with child/children	154	36.5
Sex of the smallest child in the households	Male	195	46.2
	Female	227	53.8
Age of the smallest child in months	2–26	109	25.8
	27–36	101	23.9
	37–46	114	27.1
	47–60	98	23.2
Relationship with the child/children	Mothers	346	82.2
	Caregivers	75	17.8
Have you availed of home services provided by health professionals	Yes	89	21.1
	No	333	78.9
Have you received counseling for vaccination?	Yes	331	78.4
	No	91	21.6
Did you take regular antenatal care?	No	122	28.9
	Yes	300	71.1
What is the distance of your home from the health facility?	If it is short distance, no need of using Bajaj	98	23.2
	It needs one-step Bajaj use.	219	51.9
	It needs two or more steps of Bajaj use	105	24.9
Where did you receive information about vaccination? (more than one)	From health professionals	312	73.9
	Social media (Facebook, telegram,)	219	51.9
	Television/radio	110	26.1
	From relatives/friends	40	9.5
Workload	No	50	11.8
	Medium	73	17.3
	High	229	54.3
	Very high	70	16.6
Do you fear the side effects of vaccination?	Yes	238	56.4
	No	184	43.6
Knowledge of vaccination	Poor	160	37.9
	Good	262	62.1
Attitude toward vaccination	Negative	191	45.3
	Positive	231	54.7

### Childhood vaccination practices of mothers/caregivers

**Figure 2:**
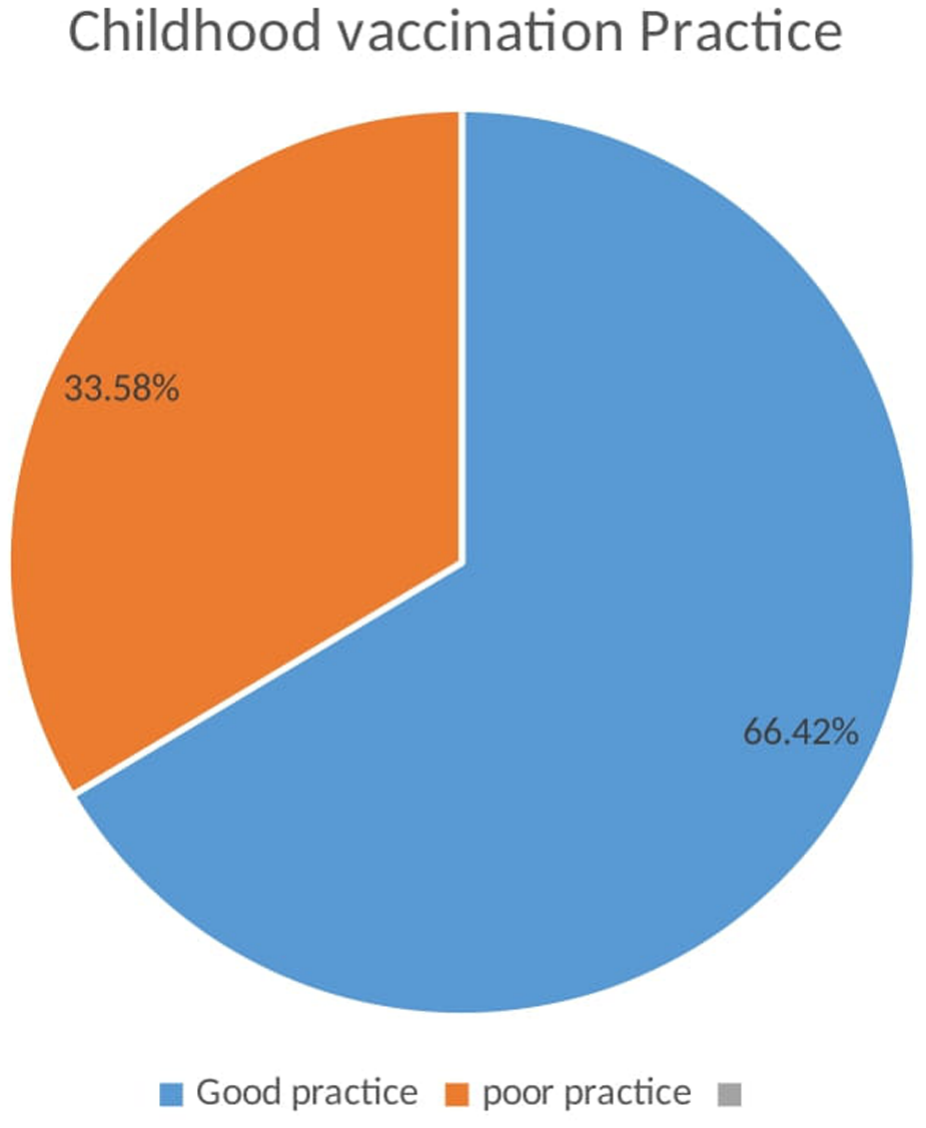
Childhood vaccination practices among mothers/caregivers in Debre Tabor, Northwest Ethiopia, 2022 (*n* = 422).

In this study, 280 (66.4%, 95% CI: 61.8–70.6) mothers/caregivers had good childhood immunization practices (Figure 2).

### Associated factors of childhood vaccination practice

Age, workload, fear of side effects if any, whether being a mother or caregiver, attitude, and knowledge were candidate variables for the final model and included in multivariable logistic regression. In the final model, fear of side effects [adjusted odds ratio (AOR) = 3.34; 95% CI: 1.72–6.49], no workload (AOR = 6.08; 95% CI: 1.74–21.22), a medium workload (AOR = 4.80; 95% CI: 1.57–14.71), being a mother of child/children (AOR = 2.55; 95% CI: 1.27–5.13), positive attitude (AOR = 2.25; 95% CI: 1.32–3.82), and good knowledge (AOR = 3.88; 95% CI: 2.26–6.68) were significantly associated with childhood vaccination practices (Table 3).

**Table 3:** Associated factors of childhood vaccination practices among mothers/care givers in Debre Tabor, Northwest Ethiopia, 2022 (*n* = 422).

Variables	Categories	Childhood vaccination practice		COR (95% CI)	AOR (95% CI)
		Good	Poor		
Age	18–27	82 (77.4)	24 (22.6)	2.27 (1.24–4.17)	1.53 (0.70–3.35)
	28–33	75 (68.8)	34 (31.2)	1.47 (0.83–2.59)	1.29 (0.60–2.78)
	34–42	63 (58.9)	44 (41.1)	0.95 (0.54–1.66)	1.08 (0.51–2.29)
	43–80	60 (60.0)	40 (40.0)	1	1
Do you fear the side effects of vaccination?	Yes	204 (85.5)	34 (14.3)	8.52 (5.34–13.59)	3.34 (1.72–6.49)**
	No	76 (41.3)	108 (58.7)		
Workload	No workload	45 (90.0)	5 (10.0)	3.11 (1.07–9.06)	6.08 (1.74–21.22)*
	Medium workload	68 (93.2)	5 (6.8)	4.70 (1.64–13.51)	4.80 (1.57–14.71)*
	High workload	115 (50.2)	114 (49.8)	0.34 (0.19–0.63)	1.35 (0.61–3.00)
	Very high workload	52 (74.3)	18 (25.7)	1	1
Relationship with the child/children?	Mother	253 (73.1)	93 (26.9)	4.83 (2.85–8.20)	2.55 (1.27–5.13)*
	Caregiver	27 (36.0)	48 (64.0)	1	1
Attitude toward vaccination	Negative	98 (51.3)	93 (48.7)	1	1
	Positive	182 (78.8)	49 (21.2)	3.52 (2.03–5.38)	2.25 (1.32–3.82)**
Knowledge of vaccination	Poor	61 (38.1)	99 (61.9)	1	1
	Good	219 (83.6)	43 (16.4)	8.26 (5.23–13.05)	3.88 (2.26–6.68)*

AOR, adjusted odds ratio; COR, crud odds ratio; CI, confidence interval.

Hosmer and Lemeshow test = 0.662.

* *<0.05;* **<0.005.

## Discussion

Immunizing children against vaccine-preventable diseases is an important public health intervention strategy to reduce the morbidity and mortality associated with infectious diseases (29). The authors suggest that an unbroken assessment of the childhood vaccination practices of mothers and caregivers would greatly help health administrators to devise immediate action plans. Following this method, this study assessed the childhood vaccination practices among mothers and caregivers and associated factors in Debre Tabor town, Northwest Ethiopia. In our investigation, we found that all children had received the first round of vaccinations. But the investigator could not trace the details of the completion of childhood vaccinations as the study included even a child at the age of 2 months.

This study found that approximately two-third of mothers and caregivers (66.4%, 95% CI: 61.8–70.6) had good childhood immunization practices. This rate was higher than that of the previous study conducted in Wadla Woreda, Northeast Ethiopia (55.3%) (26). This could be attributed to differences in study settings—the current study was conducted in a town with somewhat better educated mothers and caregivers, whereas the previous one was conducted in a rural setting. This study’s rate of finding is also lower than that of a study conducted in Addis Ababa (84%) (16). Variations in terms of information, health education, and health service accessibility would make a significant difference in the level of childhood vaccination practices. Such differences showed the existence of different kinds of health service approachability across the country for preventive care services and stressed the necessity of childhood vaccination (13). The current rate of finding also showed a higher level of practice as compared to the study conducted in Lebanon (32.8%) (30), and it is lower than that of the studies conducted in Sudan (89.5%) (29), in Saudi Arabia (80.5%) (28), and in Indonesia (71.8%) (24). The variations might be due to differences in sociodemographic characteristics, sample size, study setting, and the healthcare system of the country.

In this study, more than half of the participants (56.4%) reported fears of vaccination side effects, and 91 (21.6%) respondents missed their vaccination schedules because of the fear of side effects. In a previous study in Ethiopia, the fear of side effects was reported as one of the major reasons for the delay in childhood vaccination (13). This might be due to the fact that the side effects of vaccination were not understood by the participants, as explained in the previous study (24). This would require the creation of awareness about vaccination’s possible side effects and their overall benefits (31).

This study revealed that all study participants had proper access to information about childhood vaccination; a majority of the participants (73.9%) accessed info from health professionals, and approximately half (51.9%) reported using social media. This was similar to that of previous research in which health professionals were the major source of information, followed by social media (16, 29, 32). A study conducted in Cyprus reported that pediatricians were the major source of information as compared to other health professional specialists (33), while the present study did not investigate particular health professionals who had a greater role to play in sharing information with mothers or caregivers. The current rate of finding is higher than that of the previous local study, which found that 80.4% of mothers had heard about infant vaccination, of which 57.7% used health workers as the major source of information (26). The possible reason for this might be that the present study was conducted in a town where all people gained easy access to health professionals and social media, while the previous study was conducted in a rural area (woreda level). Another reason might be that all mothers are currently delivered in the health facility, which would help them to get information from that facility. This finding rate is also higher than that of Saudi Arabia, where 60.1% of the population received information about vaccination from healthcare professionals (28). Electronic and mass media are also creating awareness about vaccination through advertisements and special programs (34). Of course, social media may exercise a considerable (negative or positive) impact on the knowledge levels of mothers (28).

In this study, fear of side effects, workload, being a mother of child/children, attitudes toward childhood vaccination, and knowledge of childhood vaccination were associated factors for childhood immunization practices. Study participants who reported having a fear of side effects had more than three times better practices than those who reported having a fear of the side effects of childhood immunization. Several studies have also found that fearing the side effects of a vaccine had a negative impact on childhood vaccination practices (13, 29, 35, 36). The reason for this might be a poor understanding of the side effects associated with vaccination (24). This might require improving the levels of communication between health professionals and mothers/caregivers, which would play an important role in shaping parents’ beliefs by informing them about the benefits and safety of vaccines and thereby building trust in childhood vaccination (30). This study identified the impact of workload on childhood vaccination practices. Study participants who had no workload and those with a medium workload had approximately six and five times better child vaccination practices, respectively, as compared to those who reported having a higher workload. This is finding is supported by the previous study in Ethiopia (13) and another study in Pakistan (35). A qualitative study in Burkina Faso also reported the impact of workload on childhood vaccination practices (36).

This study showed being a mother of a child/children had more than 2.5 times the odds of better practices of childhood vaccination than those who were caregivers (not mothers). No other study reported a significant relationship between being a mother and childhood vaccination. The possible reason for this might be the difference in the levels of trust in vaccination among the two groups (37).

In this study, participants with a positive attitude toward childhood vaccination had more than two times better childhood vaccination practices than their negative-attitude counterparts. This finding is supported by previous studies (24, 30). Therefore, it is not surprising that a positive attitude would lead to improved and sound childhood vaccination practices (38).

Reaffirming the previous research work in Ethiopia (26), the present study participants with good knowledge of childhood vaccination had more than two times good childhood vaccination practices than others. This finding is also supported by that of previous studies conducted elsewhere (24, 29, 30, 39). Better awareness and improved knowledge about the benefits of immunization influence health-seeking behaviors (33, 34). This situation is associated with the role of primary health centers in running a routine education program for mothers and caregivers about children’s health (24). Another study revealed that the correlation between knowledge and practice of mothers was statistically insignificant (29). In contrast to the previous study (30, 33, 40–42), economic status was not significantly associated with practice in the present study. Similarly, the absence of a significant relationship between these two factors was also reported in Addis Ababa (16). The reason for this might be that immunization services are provided free of cost in public healthcare facilities all over Ethiopia. However, in Lebanon, there is no universal health coverage for immunization.

### Strengths and limitations of the study

This study assessed childhood vaccination practices and predictors showing the current situation in the study setting and used these as inputs to design interventions by stakeholders. The study had the following limitations: it included participants who were infants whose starting age was as low as 2 months, making it impossible to provide the status of full vaccination. A recall bias, the inability to determine cause and effect relationships because of the cross-sectional nature of the study, a smaller sample size that did not consider the design effect, and a single study area could all be considered the limitations of this study.

## Conclusion

This study found that approximately two-third of mothers and caregivers had good childhood vaccination practices. However, this is figure is still not up to the mark,, necessitating better design interventions. Being busy at work was reported as the major reason for parents missing their vaccination schedules. Mothers’ and caregivers’ workload, fear of side effects, motherhood, attitude toward childhood vaccination, and knowledge of childhood vaccination were all linked to childhood immunization practices. Awareness creation and considering the workload of mothers and caregivers would be helpful in promoting good childhood vaccination practices.

## Data Availability

The raw data supporting the conclusions of this article will be made available by the authors, without undue reservation.
